# Molecular Heterogeneity and Cellular Diversity: Implications for Precision Treatment in Medulloblastoma

**DOI:** 10.3390/cancers12030643

**Published:** 2020-03-10

**Authors:** Han Zou, Brad Poore, Alberto Broniscer, Ian F. Pollack, Baoli Hu

**Affiliations:** 1 Department of Neurological Surgery, University of Pittsburgh School of Medicine, Pittsburgh, PA 15213, USA; HANZOU@pitt.edu (H.Z.); POLLACI@upmc.edu (I.F.P.); 2 Pediatric Neurosurgery, UPMC Children’s Hospital of Pittsburgh, Pittsburgh, PA 15224, USA; 3 Xiangya School of Medicine, Central South University, Changsha 410013, China; 4 Department of Pathology, Dartmouth Hitchcock Medical Center, Lebanon, NH 03766, USA; Bradley.A.Poore@hitchcock.org; 5 Pediatric Neuro-Oncology Program, UPMC Children’s Hospital of Pittsburgh, Pittsburgh, PA 15224, USA; alberto.broniscer@chp.edu; 6 Molecular and Cellular Cancer Biology Program, UPMC Hillman Cancer Center, Pittsburgh, PA 15232, USA

**Keywords:** medulloblastoma, molecular subgroups, genetic and epigenetic heterogeneity, intertumoral diversity, clinical trials

## Abstract

Medulloblastoma, the most common pediatric malignant brain tumor, continues to have a high rate of morbidity and mortality in childhood. Recent advances in cancer genomics, single-cell sequencing, and sophisticated tumor models have revolutionized the characterization and stratification of medulloblastoma. In this review, we discuss heterogeneity associated with four major subgroups of medulloblastoma (WNT, SHH, Group 3, and Group 4) on the molecular and cellular levels, including histological features, genetic and epigenetic alterations, proteomic landscape, cell-of-origin, tumor microenvironment, and therapeutic approaches. The intratumoral molecular heterogeneity and intertumoral cellular diversity clearly underlie the divergent biology and clinical behavior of these lesions and highlight the future role of precision treatment in this devastating brain tumor in children.

## 1. Introduction 

Medulloblastoma (MB) is the most common malignant brain tumor of childhood, which is classified as an embryonal tumor located in the cerebellum. With an incidence rate of 0.156 cases per 100,000 population, MB ranks second behind leukemia in incidence, but carries a much worse overall prognosis [1]. Histologically and genomically, MB is a heterogeneous disease that differs greatly among patients. The histologic classification of MB consists of four types based on morphological evaluation, including Classic (CLA), desmoplastic/nodular (DN), MB with extensive nodularity (MBEN), and large cell/anaplastic (LCA) [2]. Of these findings, moderate to extensive anaplasia, along with presence of metastases, were characterized with having the worse prognosis [3,4]. Given that histological classifications can only partially reflect disease heterogeneity and insufficiently predict patient outcome, MB has since become subclassified on key molecular variations in addition to their histological characterization.

Multiple studies using genetic and transcriptional profiling of MB samples identified four distinct molecular subgroups: wingless (WNT), sonic hedgehog (SHH), Group 3, and Group 4 [5,6,7,8,9,10]. Each of these subgroups has different molecular drivers, clinical characteristics, and prognoses; for example, the SHH tumors, especially those tumors with *TP53* mutations, and Group 3 have the worse prognosis while the WNT driven MBs are associated with better prognosis [11,12]. Due to the differences in aggressiveness between the groups, the WNT-driven MB may be treated less aggressively than those with SHH or Group 3 tumors. This is important as the standard of care use of radiation therapy carries significant comorbidities, such as developmental delays or secondary cancers that can occur later in life [13,14,15]. This makes molecular classification of MBs, especially at initial diagnosis, an imperative. Therefore, the revised 2016 World Health Organization (WHO) classification requires both histological and genetical evaluation as a standard diagnosis for MB [16].

Recently, single-cell RNA sequencing (scRNA-seq) based studies provided new insights on molecular and cellular heterogeneity, which underlie the divergent biology and clinical behavior in MB [17,18,19]. This review is meant to explore the intratumoral and intertumoral heterogeneity and diversity that characterize MB, and how the differences between the subgroups could potentially contribute to the treatment and/or prognosis of affected patients.

## 2. Molecular Heterogeneity in MB 

### 2.1. Molecular Stratifications of MB

**Figure 1:**
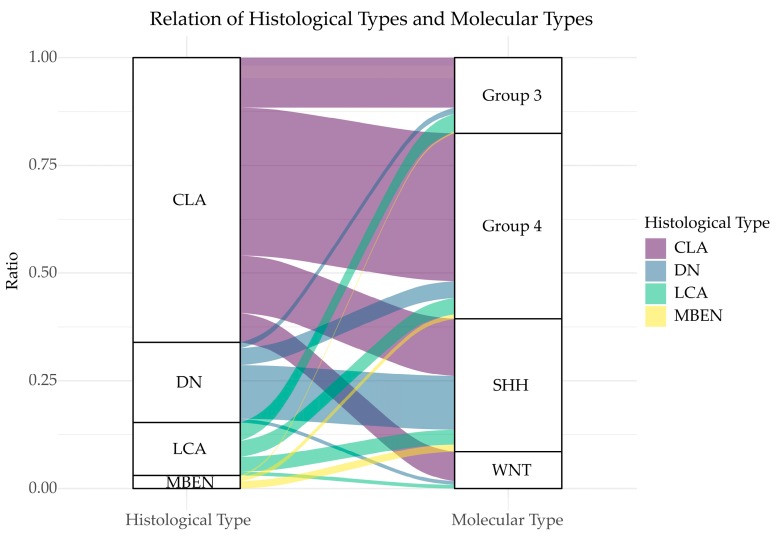
Relation of histological types and molecular types. The two columns represent histological classification and molecular classification, respectively. Different heights correspond to different ratios. Lines between the columns represent the overlapping classification systems. The broader a line, the more overlapping patients it has. This figure was made based on the date from the reference [12]. CLA: classic medulloblastoma; DN: desmoplastic/nodular medulloblastoma; LCA: large cell/anaplastic medulloblastoma; MBEN: medulloblastoma with extensive nodularity.

**Figure 2:**
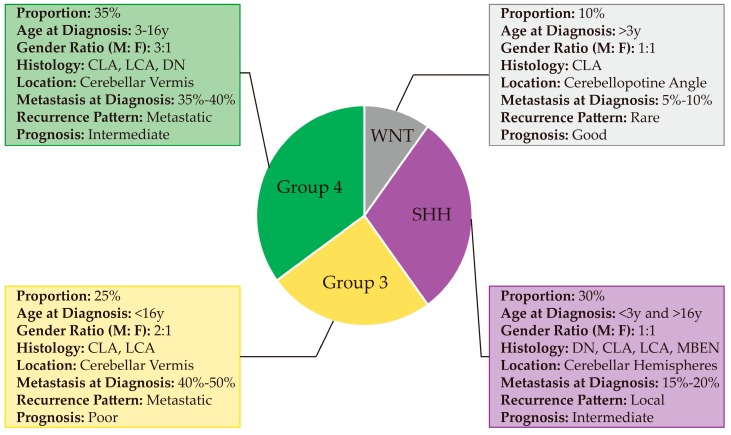
Clinical characteristics of MB subgroups. Pie chart illustrating the frequency, age, gender, and clinical features of the four subgroups of MB. The figure was made based on data from the following references [10,21,55,56,57].

In 2012, researchers reached a consensus that classified MB into four different subgroups based on their molecular characterization, namely WNT, SHH, Group 3, and Group 4 [10]. Of these subgroups, WNT and SHH were distinguished by the signaling pathways that contribute to their pathogenesis while Group 3 and Group 4 are separated based on clustering algorithms rather than a single activated pathway. WNT and SHH subgroups have a balanced sex ratio, while Group 3 and Group 4 MBs have a male predominance [10]. It is important to note that these classifications are distinct from the histological subgroupings, although there is significant overlap (Figure 1). For example, Group 3 MBs generally display a classic phenotype, although with some patients exhibiting anaplastic/large cells pathology as well [10]. Clinical features in MB subgroups were summarized in Figure 2; cellular, genetic and molecular characteristics of MB subgroups were described below and summarized in Table 1.

#### 2.1.1. WNT

Of the different subgroups, the WNT MB has the best prognosis and accounts for 10% of all patients with MB. Patients with WNT MB are expected to have > 90% survival [10,25]. Named by its core molecular pathway, WNT MB contains frequent mutations in the WNT pathway, including *CTNNB1*, deletion of chromosome 6, and strong immunohistochemical nuclear staining for β-catenin [10]. Integrated analysis of gene expression and DNA methylation further defined two WNT subtypes: WNTα and WNTβ [12]. While WNTα and WNTβ tumors have similar survival, the WNTα subtype is enriched for children who have the high frequency of monosomy 6 while WNTβ subtype primarily incorporates older children and adults with a low frequency of monosomy 6 [12].

#### 2.1.2. SHH

SHH MB is the dominant subgroup in both young children (≤3 years old) and adults (≥16 years old) [26]. This subgroup accounts for about 30% of all patients with MB, and is defined by its activation or mutation in the SHH signaling pathway, thus giving rise to the SHH nomenclature [27]. SHH tumors often contain mutations in genes that activate or promote SHH signaling, such as *PTCH1*, *SMO*, *SUFU*, and amplifications of *GLI1* and *GLI2* [27,28]. It is thought that overactivation of SHH pathway is potentially the source of tumorigenesis, as patients with germline mutations in *SUFU*, are at increased risk to develop SHH MB in infancy [28,29]. This SHH subgroup carries with it an intermediate prognosis except in instances where the tumor also harbors TP53 mutations, in which there is a poorer prognosis [30]. Additionally, infant and adult SHH MBs are distinct both clinically and molecularly [27]. Clinically, metastasis in adult SHH MBs often portends a poor prognosis, while not in young children [27]. Desmoplastic SHH MB is a mark of good prognosis in young children, but not in adults [27]. Cytogenetically, over-representation of chromosome 10q deletion and *MYCN* amplification are more significant in young children [27]. Chromosome 10q deletion, 2 gain, 17p deletion, 17q gain, and/or *GLI2* amplification in adults often means a much worse prognosis than in young children [27]. Recently, SHH MB was further classified into four subtypes: SHHα, SHHβ, SHHγ, and SHHδ based on DNA methylation and gene expression array datasets [12]. SHHα subtype is enriched for children who have frequent *TP53* mutations and *MYCN*/*GLI2* amplifications. SHHβ and SHHγ occur in young children, whereas SHHβ tumors have a worse overall survival with frequent metastases compared to SHHγ tumors that are enriched for the MBEN histology. SHHδ tumors primarily occur in adults, have a favorable prognosis and have a high frequency of *TERT* promoter mutations.

#### 2.1.3. Group 3

Of the subgroups, Group 3 has the worst prognosis in MB, with a 5-year survival ranging from 39% to 58%, depending on age of the patient and treatment regimen [26]. One potential reason for the poorer prognosis is that 50% of patients with Group 3 MB have metastases at the time of diagnosis [31,32]. The most common cytogenetic event in Group 3 is isochromosome 17q (40–50%). Other common events include loss of chromosomes 8, 10q, and 16q and gain of 1q, 7, and 18 [20]. Currently, there is no consensus if these tumors are driven by a distinct pathway, however Group 3 tumors contain recurrent *MYC* amplifications, *GABRA5* overexpression, and *SMARCA4* mutations [31]. Due to a lack of a single unifying mutation or activated pathway, these tumors are often clustered based on their transcriptional profile rather than a single marker [10,24]. A recent study based on the integrated analysis of gene expression and DNA methylation defined three subtypes of Group 3 MB: Group 3α, Group 3β, and Group 3γ [12]. Group 3α tumors are enriched for young children, while Group 3β and Group 3γ tumors occur more commonly in older children. Interestingly, Groups 3α and 3β have a more favorable prognosis compared with Group 3γ, but Group 3α and Group 3γ are more frequently metastatic compared with Group 3β. Molecularly, chromosome 8q loss is more frequent in Group 3α and gain more frequent in Group 3γ. Furthermore, *MYC* amplification is more frequent in Group 3γ; Group 3β tumors have a higher frequency of *OTX2* gain, *DDX31* loss, and high *GFI1*/*GFI1B* expression [12].

#### 2.1.4. Group 4

Group 4 MB is the most common form of MB and accounts for 35–40% of all MBs [10]. Similar to Group 3, Group 4 MB does not have a unifying molecular signature and instead must be distinguished based on the overall transcriptional/molecular profile [10]. Genetically and transcriptionally, the highly prevalent putative driver events in Group 4 involve overexpression of *PRDM6* (17%) and *GFI1*/*GFI1B* (5–10%), somatic mutations of *KDM6A* (9% ), *ZMYM3* (6%), *KMT2C* (6%) and *KBTBD4* (6%), and amplifications of *MYCN* (6%), *OTX2* (6%), and *CDK6* (6%) [20]. Cytogenetically, Group 4 tumors have the most common aberration with isochromosome 17q (80%) and other less frequent aberrations including gain of chromosomes 7 and 18q, and loss of 8q, 8p, 11p, and X [10,20]. Recently, three subtypes of Group 4 tumors were defined as Group 4α, Group 4β and Group 4γ [12]. Clinically, there is no statistically significant difference in the overall survival and metastasis rate at diagnosis between these groups. Molecular features associated with these three subtypes include *MYCN* and *CDK6* amplification in group 4α, *SNCAIP* duplication in group 4β, and *CDK6* amplification in group 4γ [12].

### 2.2. Epigenetic Regulation in MB Subgroups

Epigenetic regulation plays an important role in MB development, which mainly includes DNA methylation, histone modifications, ATP-dependent chromatin remodeling, and genomic structural variations. Importantly, epigenetic regulators serve as oncogenes or tumor suppressors in a context-dependent manner across the distinct subtype of MB [33,34,35].

#### 2.2.1. DNA Methylation

DNA methylation is a well-characterized epigenetic mechanism, typically occurring on CpG islands of gene promoter regions, resulting in transcriptional repression during normal development and tumorigenesis [36]. Based on whole genome bisulfite sequencing on 230 MB samples, Schwalbe et al. previously demonstrated that subgroups classified by DNA methylation status are highly related to their transcriptomic counterparts, indicating heterogeneity of DNA methylation associated with distinct molecular, clinical and pathological disease characteristics in MB [37]. Mechanistically, in contrast to the classical notion of gene repression though promoter hypermethylation, a comprehensive analysis by combining whole-genome, RNA, chromatin immunoprecipitation (ChIP) and whole-genome bisulphite sequencing data revealed that hypomethylation of non-promoter regions correlates with increased gene expression in MB subgroups [38]. For example, the low-risk Group 3 MB was defined primarily by hypermethylation with respect to normal cerebellum, whereas the high-risk Group 3 MB was defined by hypomethylation [39]. These studies further demonstrated complexity and diversity of DNA methylation features in MB subgroups.

#### 2.2.2. Histone Modifications

Histone modifications play a crucial role in controlling chromatin structure and gene transcription, which include histone methylation, phosphorylation, acetylation, and ubiquitination. Genomics studies of MB provided strong evidence that alterations of histone modifiers result in deregulating the epigenetic machinery, particularly in modifications of lysine methylation and/or acetylation, which fundamentally contributes to MB development in the distinct subgroups [20,40,41]. Specifically, frequent mutations of *MLL2*/*KMT2D* and *MLL3*/*KMT2C*, two histone-lysine N-methyltransferases that regulate H3K4 methylation, were identified in 16% of MB [41]. Interestingly, *MLL2* mutations were slightly enriched in WNT and SHH subgroups, while *MLL3* were found only in Group 3 and Group 4 MBs [41,42]. In contrast, *KDM6A*/*UTX*, a H3K27me demethylase binding to *MLL2*/*3* complex, is the most frequently mutated gene and co-occurs with *ZMYM3* mutations in Group 4 MBs [42,43,44]. However, EZH2, the major subunit of the H3K27 methyltransferase PRC2 complex, was identified to be highly expressed in Group 3 and Group 4 MBs with globally elevated H3K27me3 levels and a worse prognosis compared with WNT and SHH MBs [34,44]. In addition to histone methylation, histone acetylation also plays many fundamental and context-dependent roles in MB. Gene mutations of *CREBBP* and *EP300,* encoding histone acetyltransferases (HATs) CBP and p300 respectively, were found in MB [44,45,46]; these HATs catalyze H3K27ac, an active enhancer mark associated with the higher activation of gene transcription [47]. Based on high-resolution chromatin immunoprecipitation with sequencing (ChIP-seq) for active enhancers (H3K27ac) in tumor samples and cell lines, Lin and colleagues found subtype-specific super-enhancers in MB transcriptional diversity [48]. These super-enhancers regulate *ALK* in WNT, *SMO* and *NTRK3* in SHH, *LMO1*, *LMO2* and *MYC* in Group 3, and *ETV4* and *PAX5* in Group 4 MBs [48]. Another class of histone modifier, the Bromodomain and Extra-Terminal Domain (BET) family proteins (BRD2, BRD3, BRD4), recognize acetylated lysine residues on euchromatin and promote transcription, epigenetically regulate *MYC* expression in Group 3 MB, suggesting therapeutic potential for this subgroup by using BET inhibitors (e.g., JQ1) [44,49,50]. In contrast, genes encoding subunits of a nuclear receptor corepressor (N-CoR) complex, e.g., *GPS2*, *BCOR* and *LDB1*, which is associated with histone deactylases (HDACs), are frequently mutated and active in SHH MB, suggesting effective response of HDACs inhibitors in the treatment of this subgroup tumors [46,47].

#### 2.2.3. ATP-Dependent Chromatin Remodeling

ATP-dependent chromatin remodeling complexes, such as switch/sucrose non-fermentable (SWI/SNF) and chromodomain helicase DNA-binding (CHD), can utilize the energy from ATP hydrolysis to reorganize chromatin structure for regulation of gene expression. Recurrent mutations in SWI/SNF family members including *SMARCA4*/*BRG1* are the most common in WNT and Group 3 MBs than those in SHH and Group 4 MBs [41,42,44]. Of interest, SWI/SNF complex has both antagonistic and synergistic roles with PRC1 and PCR2 in context-specific conditions [51,52,53], indicting significant contribution of chromatin remodeling in MB biology and treatment. Additionally, ATP-dependent chromatin remodeling enzyme *CHD7* is frequently mutated in Group 3 and Group 4 MBs [44]. Tumors with *CHD7* mutations have reduced *EZH2* expression levels [44], further supporting an antagonistic relationship between SWI/SNF and PCR2 complexes in these subgroups of MB.

#### 2.2.4. Genomic Structural Variations

Structural variations (SVs), including all structural and quantitative chromosomal rearrangements, not only contribute to the genetic diversity of the human genome, but also modulate basic mechanisms of gene regulation by altering higher-order chromatin organization [54]. Importantly, Northcott and colleagues identified diverse SV classes associated with oncogenic activation of *GFI1B* or its paralogue *GFI1* in Group 3 and Group 4 MBs [22]. Interestingly, the high diversity of SVs affects the *GFI1B* and *GFI1* locus or surrounding genomic regions, including deletions, inversions, duplications, and interchromosomal translocations. Topologically, these SVs juxtapose *GFI1* or *GFI1B* coding sequences proximal to active enhancer elements, including super-enhancers, resulting in transcriptional activation of these oncogenes and malignant transformation in Group 3 and Group 4 MBs [22].

### 2.3. Proteomics in MB Subgroups

Genomic characterization of MB has identified the genetic and epigenetic heterogenicity but struggles to define functional biological processes involved in tumorigenesis. Proteomic and phosphoproteomic analysis could provide insight into discovering active oncogenic signaling pathways and mechanisms in MB. Using quantitative (phospho)-proteomics, Forget et al. defined highly divergent posttranscriptional pathway regulation in MB subgroups in a total of 41 flash-frozen primary MB tumors, particularly in Group 3 and Group 4 MBs [58]. Specifically, this study further validated aberrant ERBB4-SRC oncogenic signaling in Group 4, indicating potential therapeutic vulnerability by using SRC kinase inhibitors in this subgroup of MB [58]. Another study of integrated RNA expression, DNA methylation and global proteomes/phospho-proteomes of 45 MB samples identified two subsets of tumors, SHHa and SHHb, suggesting a post-transcriptional heterogeneity within SHH MB. The SHHa subset has higher levels of proteins associated with mRNA processing, splicing, and transcription, MYC pathway activation, chromatin remodeling, and DNA repair; while the higher levels of proteins in the SHHb subset are linked to neuronal and neurotransmitter-like activity, glutamatergic synaptic pathway, and MAPK/ERK signaling [59]. Interestingly, in this study, post-translational modifications of MYC (phosphorylation of residues S62 and T58 of MYC) in Group 3 tumors were defined as a higher risk factor for prediction of patient outcome. This study further reported that different kinase activities are associated with distinct subtypes of MB, including enrichment of PRKDC phosphorylation in MYC-activated MB, highlighting PRKDC inhibitors in sensitizing this subset of tumors to radiation [59].

## 3. Cellular Heterogeneity in MB

### 3.1. Histological Diversity of MB

The four main histologic types of MB (CLA, DN, MBEN, and LCA) recognized by the WHO are characterized based on their histological morphology, with separations made on the grade of nodularity, desmoplasia, and anaplasia [2,3,60]. Of these subtypes, the majority of MBs are characterized as CLA, which contains small basophilic cells with a high nuclear to cytoplasm ratio. Generally, this pattern is characterized by a high mitotic index as well as apoptotic activity. DN tumors are densely packed with cells and hyperchromatic nuclei, in addition to collagen layers stratified throughout the tumor. MBEN tumors are similar to the DN subgroup; however, portions of the tumor lack the collagen zones. LCA tumors contain high numbers of mitotic and apoptotic cells, and an altered cellular morphology. However, the anaplastic MB tends to have elevated nuclear pleomorphisms while large cell MB is characterized by large circular cells with prominent nuclei [60]. 

### 3.2. Cell of Origins in MB Subgroups

Although MBs are thought to originate from primitive and undeveloped cells in the brain, the cell of origin for MB subgroups remains controversial. Most recently, three single-cell RNA sequencing (scRNA-seq) studies have provided a clearer picture of MB putative subtype-specific origins, highlighting the molecular and cellular diversity of MB across all subgroups, with the potential insights into understanding of tumor development and treatment response [17,18,19]. 

**Figure 3:**
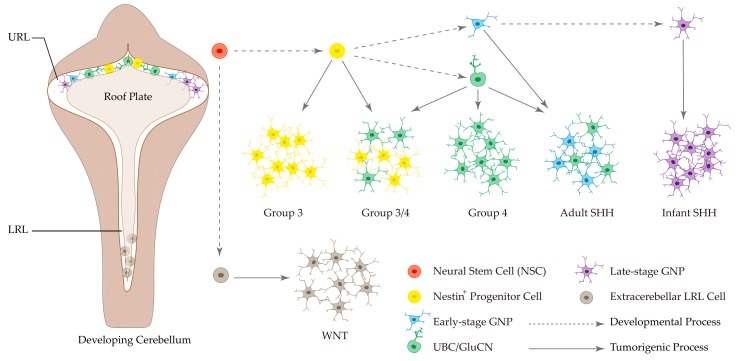
Distinct subtypes of MB originate from different progenitor cells and developmental stages. The dotted arrow represents developmental process, while the solid arrow represents tumorigenic process. Cells with same color have the same origination. This figure was made based on data from the references [17,18].

Based on investigation of associations between genotype and MB cell type, Gibson et al. discovered that WNT MBs arise outside the cerebellum from the lower rhombic lip (LRL) and embryonic dorsal brainstem, whereas SHH MBs are thought to originate from the cerebellar hemispheres [61]. Furthermore, genetically engineered mouse model studies demonstrated that SHH MBs arise from cerebellar granule neuron progenitors (GNPs) [62,63]. By single-cell transcriptomics of SHH mouse models, OLIG2^+^ glial lineage progenitors were identified to play a pivotal role in tumor initiation, therapy-resistance and recurrence [19]. Interestingly, SHH MBs in infants (≤3 years old) and adults (≥16 years old) are thought to originate from different GNP populations. Infant SHH MBs are correlated with intermediate and mature granule neurons, while adult SHH MBs are correlated with GNPs and mixed unipolar brush cells (UBCs) and GNPs [17]. In contrast to WNT and SHH MBs, cellular origins of Group 3 and Group 4 MB remain unclear. Recent scRNA-seq studies uncovered a distinct cellular hierarchy from undifferentiated to differentiated neuronal linkage in MB subgroups, particularly in Group 3 and Group 4 tumors [17,18]. Group 3 MBs are dominated by an undifferentiated progenitor-like program and thought to arise from Nestin^+^ stem cells, which give rise to a variety of differentiated progeny including GNPs and UBCs [17,18]. In contrast, Group 4 MBs are dominated by a differentiated neuronal-like program and associated with neuronal cell fates in the embryonic upper rhombic lip (URL), including UBCs and glutamatergic cerebellar nuclei (GluCN) as candidate cells-of-origin for this subgroup [17,18]. As for Group 3 and Group 4 tumors exhibiting overlapping molecular signatures, a subset of ‘intermediate’ tumors (Group 3/4) are mixed, containing both undifferentiated and more differentiated populations [17]. Together, these studies provided a clear landscape of MB subtype-specific cell-of-origin during cerebellar development (Figure 3), further supporting cellular and developmental diversity in MB biology and providing a proximate explanation for the peak incidence of MBs in childhood. It would be interesting to understand whether the originating cells (e.g., NSCs, UBCs, GNPs) are preferentially nourished in these anatomic niches for the development of each subgroup-specific MB in future research directions.

### 3.3. Diversity of Tumor Microenvironment in MB

The tumor microenvironment (TME) plays an important role in terms of tumor progression, evolution, and overall prognosis. TME encompasses the various signaling molecules, supporting cells, immune system cells, blood vessels, extracellular matrices, and nutrients that contribute to tumor progression and therapy response [64,65]. Emerging evidence based on preclinical MB models and bioinformatic analyses of clinical MB samples indicates significant TME heterogeneity across different MB subgroups [66,67,68,69].

The blood–brain barrier (BBB), an anatomic structure consisting of a variety of cell types including endothelial cells (ECs), astrocytes and pericytes, is also an important factor in maintaining TME. Of interest, there is often a functional BBB that prevents the tumor from being exposed to potential chemotherapies found in the blood stream. However, WNT MB, compared to other MB subgroups, was identified to have a paucity of functional BBB, making this subset of tumors potentially more susceptible to chemotherapies that may not cross the BBB [67]. Mechanistically, Wnt/β-catenin signaling being a necessary pathway for BBB formation is thought to be inactive in tumor surrounding ECs in WNT MB [67,70].

Infiltration of various immune cells in TME is of great interest because these infiltrating leukocytes either interfere with tumor progression or promote tumor growth, underlying response and efficacy of immunotherapy. Recent studies based on the quantification of gene expression signatures uncovered dramatical diversity of immune TME among the MB subgroups [68,69]. Of interest, SHH MB, but not Group 3 MB, displays strong signatures of macrophages and T cells, while Group 3 MB is enriched with the highest number of CD8^+^ T cells; PD-L1 expression is highest in WNT and SHH MBs, but lowest in Group 4 MB; Group 3 and Group 4 MBs have the largest number of cytotoxic lymphocytes and ECs [68]. Importantly, the study of murine SHH and Group 3 MB models further confirmed significantly higher percentages of infiltrating immune cells including tumor-associated macrophages (TAMs) in SHH tumors compared with Group 3 tumors; however, Group 3 tumors were enriched with more PD-1^+^ CD8^+^ T cells, resulting in a survival benefit in the Group 3 animals only after the treatment with PD-L1 and CTLA4 inhibitors [66]. Therefore, these TME characteristics provide promising potential of immunotherapy for treating MB. Several clinical trials have been conducting by using immune checkpoint blockade and chimeric antigen receptor T (CAR-T) cell therapies as well as therapeutic vaccines [71]. Nonetheless, it would be essential to integrate molecular and immune classification of MB for guiding future precision immunotherapy.

## 4. Diagnosis, Current Therapies and Clinical Trials for MB Subgroups

### 4.1. Diagnosis of MB Subgroups

Clinically, a physical examination aligning with neuroimaging, biopsy, and cerebrospinal fluid tests is in general being used for MB diagnosis. In 2016, the World Health Organization (WHO) classification of the Central Nervous System (CNS) tumors initiated an integrative approach including molecular parameters in combination with histology for MB diagnoses [16]. Given the many possible histological-molecular combinations, the 2016 CNS WHO presented 5 genetic variants (WNT-activated, SHH-activated/TP53-mutant, SHH-activated/TP53-wildtype, non-WNT/non-SHH/Group 3, and non-WNT/non-SHH/Group 4) in addition to the long-established histological variants (CLA, DN, MBEN, and LCA) [16]. This diagnoses approach allows greater flexibility for clinical pathologists with the ability to undertake practical methods in molecular classification. A previous study reported a NanoString 22-gene signature based on mRNA expression to stratify molecular subgroups of MB [23]. Furthermore, Gómez et al. reported a novel method for clinically applicable classification of MB based on DNA methylation detection of tumor samples [24]. Besides molecular subgrouping of MB using gene transcription and DNA methylation features, magnetic resonance imaging-based radiomic approach is a powerful tool for rapid diagnosis of MB molecular subgroups in clinic [55,72]. In addition, patient risk stratification in MB subgroups, based on age, metastatic stage, genetic and cytogenetic alterations, should be considered in diagnosis due to its significance for prognosis and treatment modalities, which was summarized in Table 2. Thus, practical and reliable biomarkers for risk stratification are important in MB diagnosis because molecular heterogeneity leads to prognostic variables in the distinct subgroups and even in the same subgroups of MB (Table 2). To this end, Shih et al. identified a small panel of cytogenetic biomarkers (GLI2, MYC, chr11, chr14, 17p, and 17q) to distinguish high-risk and low-risk patients with SHH, Group 3, and Group 4 MBs, which may provide an excellent tool in patient selection for precision therapy [73].

### 4.2. Current Therapies

The therapies for MB treatment are currently based on the patient’s risk factors, and consist of surgery, radiotherapy (RT), and chemotherapy (CT) [31,75]. Although maximal safe resection is the first-line treatment for MB, the prognostic benefit of increased extent of resection is attenuated when molecular subgroup affiliation is taken into account [76]. In a retrospective study, patients with Group 4 MB, especially those with metastatic tumor, showed the progression-free survival (PFS) benefit from gross total resection (GTR) compared to sub-total resection (STR), while this phenomenon was not observed in WNT, SHH, and Group 3 MBs [76]. However, overall survival (OS) benefit from GTR vs. STR was not observed in all subgroups of MB [76].

Craniospinal irradiation (CSI) is usually a follow-up treatment after surgery for children older than age 3 years. Based on patient risk, the treatment dose is 23.4 Gray (Gy) for standard-risk patients and 36–39 Gy for high-risk patients [77,78,79,80,81]. After the radiation, patients (>3 years of age) receive chemotherapeutic agents include vincristine, cisplatin, cyclophosphamide, lomustine, etoposide, and methotrexate [82]. For young children (<3 years of age), multi-agent chemotherapy and autologous stem cell transplantation are considered to avoid severe long-term cognitive effects from radiation [82,83]. Despite the current standard of care improves survival rates, iatrogenic morbidity and late effects often occur in children who survive MB. Therefore, new therapeutic approaches based on molecular classification must be developed to reduce these side effects for children with this brain tumor.

### 4.3. Clinical Trials

With increasing knowledge in MB genomics and biology, precision medicine is an emerging approach to clinical care that takes into account tumor genetic make-up and individual variations. Based on molecular classification of MB, we summarized the completed and ongoing clinical trials in Table 3. Current clinical trials in WNT MB are focused on decreasing the doses of RT and CT, rather than targeting WNT signaling, because this subgroup of tumor has a more permeable BBB caused by the dysfunctional WNT signaling pathway, which enables better penetration of CT molecules into cancer cells. In addition, restoration of WNT signaling activity can attenuate CT sensitivity [67,81]. There are several trials in progress and completion including lower doses of RT+CT (NCT02066220, NCT01878617, and NCT02724579) and CT-only tests (NCT02212574).

SHH MB with recurrent mutations in *PTCH1* or *SMO* can benefit from SMO inhibitor, vismodegib [84,85]. However, high-risk SHH patients harboring *SUFU* mutation, *MYCN* and *GLI2* amplifications, may not benefit from vismodegib treatment, and patients may also develop irreversible growth plate fusions after vismodegib treatment, which all limit widespread clinical application [86,87,88,89]. Therefore, there is a pressing need for new therapeutic strategies for the highest-risk groups of SHH patients. The completed and ongoing clinical trials include evaluating vismodegib alone in children and adults with refractory or recurrent SHH MB (NCT00939484, NCT01239316), oral LDE225 (Sonidegib) in relapsed SHH MB, and vismodegib in combination with temozolomide in SHH MB. Other clinical trials are ongoing, including testing CX-4945 drug (silmitasertib sodium), an orally bioavailable, highly selective and potent CK2 inhibitor, in children with recurrent SHH (NCT03904862), and fimepinostat, a synthetic, orally-available, small molecule that potently inhibits the activity of HDAC and PI3 kinase enzymes in recurrent medulloblastoma (NCT03893487). In addition, one open trial aims to assess the combination of ribociclib and sonidegib on patients with refractory or recurrent SHH at St. Jude Children’s Research Hospital (SJDAWN). 

Group 3 and Group 4 MBs have heterogenous clinical characteristics and outcomes associated with *MYC* or *MYCN* amplification, metastasis, and young age (<3 years of age). A multicenter clinical trial is ongoing in the comprehensive evaluation of current treatment options by integrating molecular subgroup and risk stratification status into the trial design (NCT01878617), in which Group 3/Group 4 MBs are prioritized for more rigorous treatment with pemetrexed and gemcitabine [21]. While *MYC* plays an important oncogenic role in many cancers, it is challenging to be directly targeted by small molecules and antibodies due to a lack of an enzymatic active site and its nuclear location [90]. Given that inhibition of BET by small molecule JQ1 resulted in suppressing *MYC* expression and thereby induce cell death [50], a pediatric cancer trial is currently underway including assessing the BET inhibitor BMS-986158 in MB with *MYC*/*MYCN* amplification (NCT03936465). Furthermore, ongoing trials aim to evaluate the inhibition of checkpoint kinases (e.g., CDK4/6, CDK1/2) alone or in combination with CT drugs in brain tumors including recurrent and refractory SHH, Group 3/Group 4 MBs (NCT02255461, NCT04023669). 

## 5. Conclusions

Recent advances in cancer genomics, single-cell sequencing, and sophisticated tumor models have revolutionized our understanding of the biology of MB. It is becoming increasingly clear that MB is a heterogeneous disease with a high degree of diversity on various molecular and cellular levels. Four major subgroups of MB (WNT, SHH, Group 3, and Group 4) display tremendous subtype-specificity in genetic and epigenetic alterations, proteomic landscape, cell-of-origin, tumor microenvironment, current therapies, and clinical trial design. Undoubtedly, these findings shed unprecedented light on the development of tailored treatment for children with MB. However, the side effects of current therapies are still a major obstacle to successful MB treatment. In the future, greater emphasis needs to be placed on the molecular characterization of MB in the clinic, as identification of the individual subgroups at diagnosis could help shape the treatment and care of the patients, and potentially improve the overall survival. Furthermore, as further elucidation of the activated pathways becomes known, precise and effective therapies targeting the driver mutations in subtype-specificity can be made available to the patients with this devastating brain tumor in children.

## Figures and Tables

**Table 1:** Summary of cellular, genetic and molecular and characteristics in MB subgroups.

	Recurrent Gene Amplification [7,10,20,21]	Recurrent SNVs [7,10,20,21]	Gain of Chromo-Some [7,10,21]	Loss of Chromo-Some [7,10,21]	Other Recurrent Genetic Events [7,10,21,22]	Signature Transcriptional Markers [23]	Signature Methylation Markers [24]	Cell of Origin [17,18]
**WNT**	NA	*CTNNB1*, *DDX3X*, *SMARCA4*, *TP53*	NA	6	NA	*WIF1*, *TNC*, *GAD1*, *DKK2*, *EMX2*	*LHX6* (cg25542041) *USP40* (cg12925355) *KIAA1549* (cg01268345)	Progenitors in LRL and dorsal brainstem
**SHH**	*MYCN*, *GLI1* or *GLI2*	*PTCH1*, *TERT*, *SUFU*, *SMO*, *TP53*	3q, 9p	9q, 10q, 17p	NA	*PDLIM3*, *EYA1*, *HHIP*, *ATOH1*, *SFRP1*	lncRNA2178 (cg02227036) *CHTF18* (cg10333416) *KIAA1549* (cg01268345)	Granule neurons (infant); GNPs and UBCs (adult)
**Group 3**	*MYC*, *MYCN*, *OTX2*	*SMARCA4*, *KBTBD4*, *CTDNEP1*, *KMT2D*	1q, 7, 18	8, 10q, 11, 16q	Isochromosome 17q; *GFI1* and *GFI1B* enhancer hijacking	*IMPG2*, *GABRA5*, *EGFL11*, *NRL*, *MAB21L2*, *NPR3*	*RPTOR* (cg09929238 and cg08129331) *RIMS2* (cg12565585) *VPS37B* (cg13548946) Intergenic region in chromosome 12 (cg05679609)	Nestin positive stem cells
**Group 4**	*SNCAIP*, *MYCN*, *OTX2*, *CDK6*	*KDM6A*, *ZMYM3*, *KTM2C*, *KBTBD4*	7, 18q	8, 11p, X	Isochromosome 17q; *PRDM6*, *GFI1*, and *GFI1B* enhancer hijacking	*KCNA1*, *EOMES*, *KHDRBS2*, *RBM24*, *UNC5D*, *OAS1*	*USP40* (cg12925355) *AKAP6* (cg18849583) lncRNA2178 (cg02227036)	UBCs and GluCNs in URL

**Table 2:** Risk stratification of MB subgroups.

	WNT	SHH	Group 3	Group 4	Intermediate 3/4 Group
**Low Risk (>90% survival)**	<16 years (age)		Chromosome 13 loss without neither *MYC* amplification nor metastasis	Non-metastatic, and whole chromosome 11 loss or whole chromosome 17 gain	All
**Average (standard) (75–90% survival)**		*TP53* wildtype without metastasis and *MYCN* amplification	Neither metastasis nor *MYC* amplification	Neither metastasis nor chromosome 11 loss	
**High Risk (50–75% survival)**		Metastatic, and/or *MYCN*-amplified		Metastatic	
**Very High Risk (<50% survival)**		Adult with *TP53* mutation	Metastatic or *MYC* amplification		

This table was made based on data from the following references [11,21,39,74].

**Table 3:** Clinical trials targeting different medulloblastoma groups.

Conditions	Interventions	ClinicalTrials.gov Identifier	Status
WNT	Surgery + Reduced-Dose Radiotherapy + Reduced-Dose Chemotherapy	NCT02066220 NCT01878617 NCT02724579	Recruiting
WNT	Surgery + Chemotherapy, No Radiotherapy	NCT02212574	Suspended
Targeting SHH pathway	Vismodegib (SMO Inhibitor)	NCT00939484 NCT01239316	Completed
Targeting SHH pathway	Vismodegib in combination with Temozolomide	NCT01601184	Terminated
Targeting SHH pathway	Sonidegib (SMO Inhibitor)	NCT01708174	Completed
Targeting SHH pathway	CX-4945 (CK2 Inhibitor)	NCT03904862	Recruiting
Intensified Treatment of Group 3/Group 4 MB	Pemetrexed and Gemcitabine	NCT01878617	Recruiting
MYC-driven Group 3 MB	BMS-986158(Bromodomain (BRD) and Extra-Terminal Domain (BET) Inhibitor	NCT03936465	Recruiting
Group 3 MB	PD-0332991/Palbociclib (CDK 4-6 Inhibitor)	NCT02255461	Terminated
Refractory or Recurrent Group 3/Group 4 MB	Prexasertib (CHK1/2 Inhibitor) and Gemcitabine	NCT04023669	Recruiting
Refractory or Recurrent SHH, Group 3/Group 4 MB	Prexasertib (CHK1/2 Inhibitor) and Cyclophosphamide	NCT04023669	Recruiting
Recurrent MB	Fimepinostat (HDAC and PI3K inhibitor)	NCT03893487	Recruiting
Refractory or Recurrent SHH MB	Ribociclib and Sonidegib	SJDAWN	Recruiting
